# Admission blood glucose and 30-day mortality in patients with acute decompensated heart failure: prognostic significance in individuals with and without diabetes

**DOI:** 10.3389/fendo.2024.1403452

**Published:** 2024-07-05

**Authors:** Jing Hu, Hongyi Yang, Meng Yu, Changhui Yu, Jiajun Qiu, Guobo Xie, Guotai Sheng, Maobin Kuang, Yang Zou

**Affiliations:** ^1^ Department of Cardiology, Jiangxi Provincial People’s Hospital, The First Affiliated Hospital of Nanchang Medical College, Nanchang, China; ^2^ Department of Ultrasound, the Second Affiliated Hospital of Nanchang University, Nanchang, China; ^3^ Jiangxi Cardiovascular Research Institute, Jiangxi Provincial People’s Hospital, The First Affiliated Hospital of Nanchang Medical College, Nanchang, China

**Keywords:** admission blood glucose, acute decompensated heart failure, diabetes, heart failure, poor prognoses

## Abstract

**Objective:**

Diabetes is a significant risk factor for acute heart failure, associated with an increased risk of mortality. This study aims to analyze the prognostic significance of admission blood glucose (ABG) on 30-day mortality in Chinese patients with acute decompensated heart failure (ADHF), with or without diabetes.

**Methods:**

This retrospective study included 1,462 participants from the JX-ADHF1 cohort established between January 2019 to December 2022. We conducted multivariate cox regression, restricted cubic spline, receiver operating characteristic curve analysis, and mediation analysis to explore the association and potential mechanistic pathways (inflammation, oxidative stress, and nutrition) between ABG and 30-day mortality in ADHF patients, with and without diabetes.

**Results:**

During the 30-day follow-up, we recorded 20 (5.36%) deaths in diabetic subjects and 33 (3.03%) in non-diabetics. Multivariate Cox regression revealed that ABG was independently associated with 30-day mortality in ADHF patients, with a stronger association in diabetics than non-diabetics (hazard ratio: Model 1: 1.71 vs 1.16; Model 2: 1.26 vs 1.19; Model 3: 1.65 vs 1.37; Model 4: 1.76 vs 1.33). Further restricted cubic spline analysis indicated a U-shaped relationship between ABG and 30-day mortality in non-diabetic ADHF patients (*P* for non-linearity < 0.001), with the lowest risk at ABG levels approximately between 5-7 mmol/L. Additionally, receiver operating characteristic analysis demonstrated that ABG had a higher predictive accuracy for 30-day mortality in diabetics (area under curve = 0.8751), with an optimal threshold of 13.95mmol/L. Finally, mediation analysis indicated a significant role of inflammation in ABG-related 30-day mortality in ADHF, accounting for 11.15% and 8.77% of the effect in diabetics and non-diabetics, respectively (*P*-value of proportion mediate < 0.05).

**Conclusion:**

Our study confirms that ABG is a vital indicator for assessing and predicting 30-day mortality risk in ADHF patients with diabetes. For ADHF patients, both with and without diabetes, our evidence suggests that physicians should be alert and closely monitor any changes in patient conditions when ABG exceeds 13.95 mmol/L for those with diabetes and 7.05 mmol/L for those without. Timely adjustments in therapeutic strategies, including endocrine and anti-inflammatory treatments, are advisable.

## Introduction

Acute decompensated heart failure (ADHF) is one of the most common reasons for hospitalization or the need for urgent care in the elderly population, posing a significant public health issue globally (1, 2). Despite tangible advances in the discovery of new heart failure treatment methods over the past few decades, the improvement in prognosis for ADHF patients during the acute phase remains limited, as these individuals still face high risks of in-hospital mortality and readmission (2–6).

Stress hyperglycemia refers to the acute increase in blood glucose during the acute phase of an illness, typically returning to pre-attack levels upon recovery (7). Admission blood glucose (ABG) is a commonly used indicator for assessing stress hyperglycemia and has been reported in numerous studies (8–15). Overall, ABG is an important marker for assessing the prognosis of acute onset diseases and severe illnesses, both in diabetic and non-diabetic populations. ADHF, as an acute exacerbation of heart failure, shows that approximately half of the acute heart failure (AHF) patients experience elevated ABG (16, 17). However, it is noteworthy that several published studies have shown contradictory correlations between ABG and short-term adverse outcomes, such as 30-day mortality, in AHF patients (16–18). Moreover, there is currently a lack of research data on the correlation between ABG and ADHF prognosis based on the Chinese population. In this context, our aim is to further evaluate the predictive significance of ABG on 30-day mortality in ADHF patients, both with and without diabetes, through a study cohort in Jiangxi, China.

## Methods

### Study population and design

**Figure 1:**
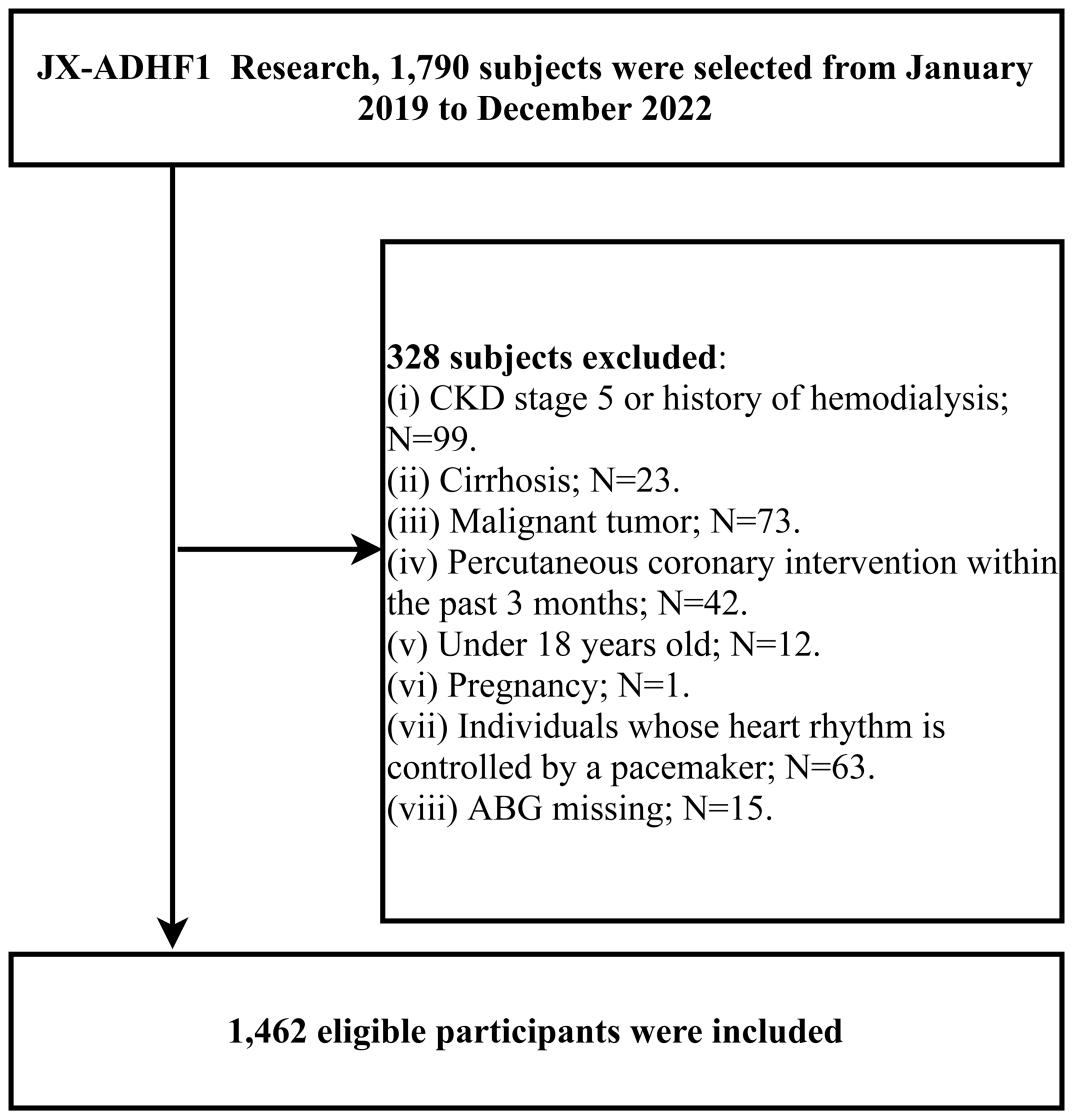
Flow chart of study participants.

The JX-ADHF1 (Jiangxi-acute decompensated heart failure1) study is a retrospective cohort study that consecutively enrolled 1,790 patients with ADHF who were treated at Jiangxi Provincial People’s Hospital from January 2019 to December 2022. The aim was to explore significant factors affecting short-term adverse outcomes in ADHF patients, thereby facilitating improvements in ADHF treatment approaches. Exclusion criteria applied to subjects with the following baseline characteristics: (i) those with stage 5 chronic kidney disease or a history of hemodialysis (n=99), and patients with cirrhosis (n=23), considering the potential effects of other diseases on sodium and water retention (19); (ii) patients with malignant tumors, given the potential impact on life expectancy (n=73); (iii) individuals who had undergone percutaneous coronary intervention within the past three months were excluded, due to the significant role of reperfusion therapy on short-term prognosis (n=42); (iv) participants under the age of 18 (n=12); (v) pregnant participants (n=1); and (vi) individuals with pacemaker-controlled heart rhythms, as their heart rate was not expected to be under autonomic control (n=63). Additionally, for the purposes of the current study, we further excluded participants with missing information on the independent variable ABG (n=15); ultimately, the study comprised 1,462 participants. A detailed flow chart of the study population screening process was shown in **Figure 1**.

In this study, the diagnosis of ADHF was defined according to the 2021 ESC and 2022 ACC/AHA/HFSA Guidelines for HF (20, 21), taking into account the subjects’ clinical symptoms, signs, and laboratory findings, mainly as follows: (1) At least one sign of HF: (a) Elevated N-Terminal Pro-Brain Natriuretic Peptide (NT-proBNP) or BNP; (b) pulmonary edema detected by physical examination or chest X-ray; (c) cardiac ultrasound showing structural and/or functional abnormalities of the heart. (2) Further deterioration of at least one symptom: (a) venous congestion of the body circulation; (b) dyspnea; (c) inadequate tissue perfusion.

### Ethics and approval

The JX-ADHF1 study adhered to the ethical guidelines of the Declaration of Helsinki. The use of study data was consented to by the participants and their families. The research protocol received authorization from the Ethics Review Committee of Jiangxi Provincial People’s Hospital (IRB:2024-01). The entire research process strictly followed the STROBE reporting guidelines (**Supplementary Table 1**).

### Data collection

Baseline information included general demographic data (gender and age), New York Heart Association (NYHA) functional classification at admission, comorbidities [hypertension, diabetes, cerebral infarction, coronary heart disease (CHD)], the most recent echocardiogram results, systolic and diastolic blood pressure (SBP and DBP), laboratory parameters from blood samples and information on medications received during hospitalization, including anti-HF treatment [diuretic, angiotensin-converting enzyme inhibitors (ACEI)/angiotensin receptor inhibitors (ARB)/angiotensin receptor neprilysin inhibitors (ARNI), beta-blockers, digitalis, sodium-dependent glucose transporters 2 (SGTL-2)] and corticosteroids therapy (spirolactone, adrenaline, and glucocorticoid). The past diagnoses of hypertension/diabetes/cerebral infarction/CHD were based on patients’ self-report, ongoing medication treatment, or records in patient medical history.

Blood pressure information was the first measurement recorded after admission, taken in a quiet environment or bedside using an Omron automatic blood pressure monitor (HBP-1300).

Laboratory parameters were measured within 24 hours of admission at the Jiangxi Provincial People’s Hospital Laboratory Center, including ABG, NT-proBNP, white blood cell count (WBC), red blood cell count (RBC), hemoglobin (HGB), platelet count (PLT), albumin (ALB), alanine aminotransferase (ALT), aspartate aminotransferase (AST), gamma-glutamyl transferase (GGT), creatinine (Cr), hemoglobin (HbA1c), total cholesterol (TC), triglycerides (TG), low-density lipoprotein cholesterol (LDL-C), and high-density lipoprotein cholesterol (HDL-C); liver enzyme-related parameters and lipid-related parameters were determined by venous blood draw in a fasting state at admission or the next morning after admission.

### Study outcome

The starting point for follow-up was defined as the time of admission, and the endpoint was 30 days post-admission. Follow-up was conducted by trained medical personnel through text messages, phone calls, and face-to-face visits in the outpatient/inpatient department. The primary endpoint of interest was death from any cause within 30 days.

### Statistical analysis

In this study, we summarized the baseline characteristics of the study population according to the diabetes status of the subjects and used inverse probability of treatment weighting and unpaired chi-square tests to calculate differences between groups for continuous and categorical variables. *P*-values for the assessment of differences between groups were chosen from t-tests, chi-square tests, or rank-sum tests, depending on the type and distributional characteristics of the variables. The baseline information of the subjects was recorded as mean (standard deviation), median (interquartile range), or count (percentage), based on the characteristics and distribution of the variables.

We first conducted survival analysis using Kaplan-Meier and log-rank tests to assess the survival status of ADHF patients in the diabetic and non-diabetic groups. Then, we employed multivariate Cox regression models to evaluate the association between ABG and 30-day mortality in ADHF patients with and without diabetes. The adjustment of variables considered sex, age, hypertension, diabetes, cerebral infarction, CHD, NYHA classification, SBP, DBP, left ventricular ejection fraction (LVEF), WBC, RBC, Hb, PLT, ALB, AST, GGT, Cr, TG, HDL-C, LDL-C, NT-proBNP, HbA1c, etiology of ADHF, ADHF seizure characteristics, anti-heart failure treatment, and corticosteroids therapy. ALT and TC were excluded from the model due to severe collinearity with other covariates (**Supplementary Table 2**) (22, 23). Additionally, the Schoenfeld residuals plot of ABG over time (**Supplementary Figure 1**) suggested that our model met the proportional hazards assumption (*P* = 0.863) (24, 25). Finally, to further test the robustness of our analysis of the association between ABG and 30-day mortality in ADHF patients, we calculated an E-value based on the effect size of ABG in the final model to quantify the minimum strength of association a confounder would need with the study outcome (26).

Based on Cox regression, we continued with restricted cubic splines (RCS) to fit the dose-response relationship curve between ABG and 30-day mortality. Additionally, we conducted stratified analysis by age (stratified based on median), gender, and comorbidities, with differences between strata examined by likelihood ratio tests.

To assess the predictive value of ABG for 30-day mortality in ADHF patients in diabetic and non-diabetic populations, we constructed receiver operating characteristic (ROC) curves and calculated the area under the curve (AUC), optimal threshold, sensitivity, and specificity for the ABG of both diabetic and non-diabetic groups.

Having established the longitudinal association between ABG and 30-day mortality in ADHF patients in diabetic and non-diabetic populations, we planned to further explore the roles of inflammation (27), oxidative stress (28), and nutrition (29) in ABG-related ADHF patient 30-day mortality. Based on previous reports, we chose WBC as a marker of inflammation (30), GGT as a marker of oxidative stress (30, 31), and ALB as an indicator of nutritional status (32). We constructed mediation effect models to analyze the influence of WBC, GGT, and ALB in ABG-related ADHF patient 30-day mortality in diabetic and non-diabetic populations, quantifying mediation effects by calculating the percentage of mediation (ratio of indirect effect to total effect) and using the bootstrap sampling method (times=1000) to test the significance of mediation effects (33).

In this study, a two-sided *P* < 0.05 or a standardized difference value >10% was considered statistically significant. All analyses were performed using R language version 4.2.1 and Empower(R) version 2.20 statistical software.

## Results

### Study cohort

The current study cohort included 1,462 participants, comprising 840 males (57.46%) and 622 females (42.54%), with an average age of 68 years. We identified nine common causes of HF in these study populations, including ischemic cardiomyopathy, hypertension, non-ischemic cardiomyopathy, valvular disease, arrhythmia, acute myocarditis, congenital heart disease, pulmonary heart disease and other reasons (including pericardial disease, hypothyroidism, anemic heart disease, severe infections, rapid progression of other systemic diseases, and unknown etiologies). Of note, the primary etiologies of ADHF patients in the current cohort were ischemic and nonischemic cardiomyopathy and valvular disease (**Table 1**).

**Table 1:** The most common aetiologies of acute decompensated heart failure.

Etiology of ADHF	All (N=1462)
Ischemic cardiomyopathy	435 (29.75%)
Hypertension	145 (9.92%)
Non-ischemic cardiomyopathy	354 (24.21%)
Valvular disease	302 (20.66%)
Arrhythmia	81 (5.54%)
Acute myocarditis	9 (0.62%)
Congenital heart disease	39 (2.67%)
Pulmonary heart disease	76 (5.2%)
Other reasons	21 (1.44%)

**Figure 2:**
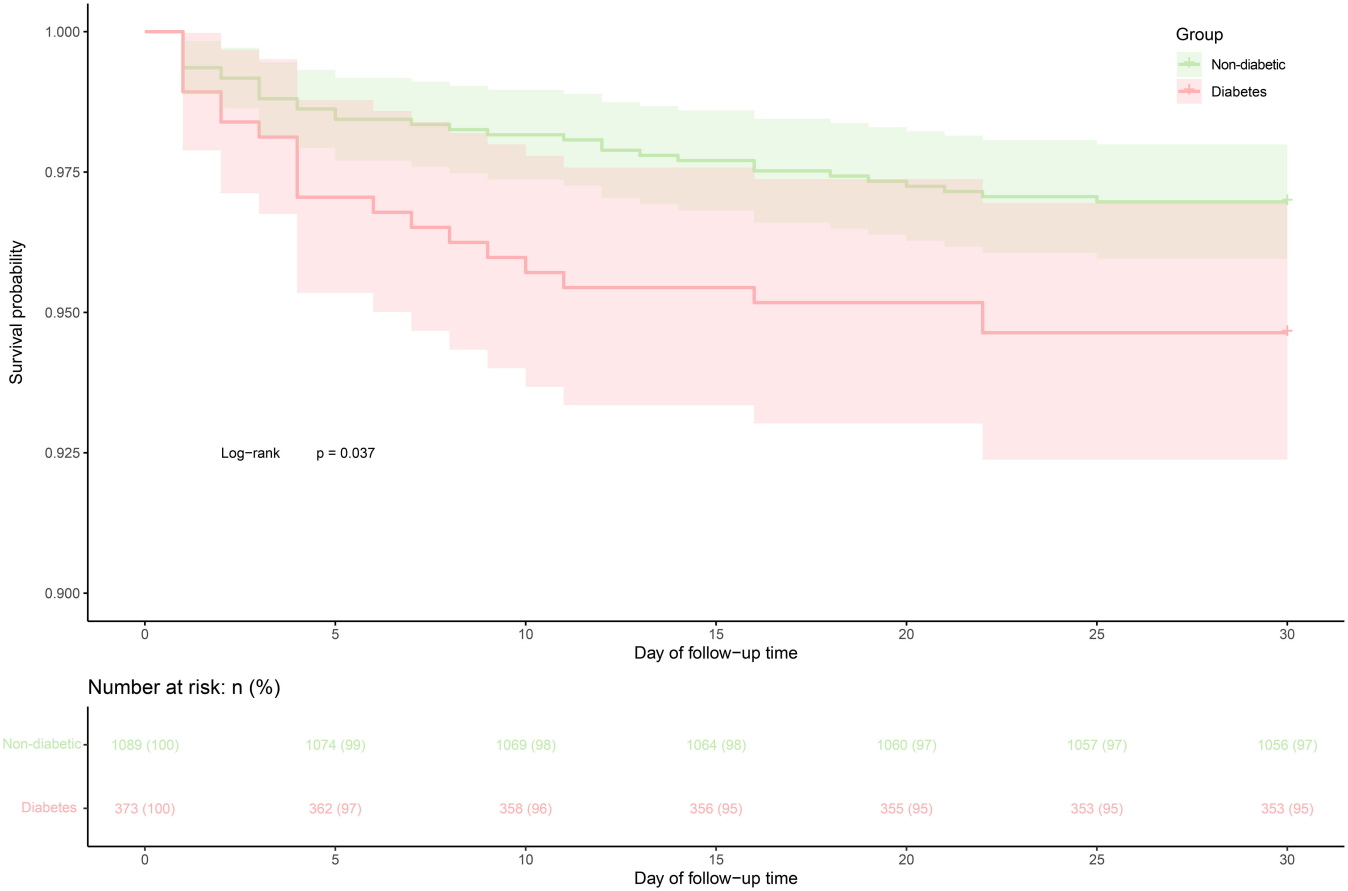
30-day survival curve of ADHF patients in diabetes and non-diabetic groups. ADHF, acute decompensated heart failure.

During the 30-day follow-up, 53 deaths (3.63%) were recorded, with a mortality rate of 5.36% (20/373) in ADHF patients with diabetes and 3.03% (33/1,089) in those without. **Figure 2** illustrates the 30-day cumulative survival curves for ADHF patients with and without diabetes, indicating significantly higher mortality within 30 days for patients with diabetes compared to those without (log-rank *P* = 0.037).

**Figure 3:**
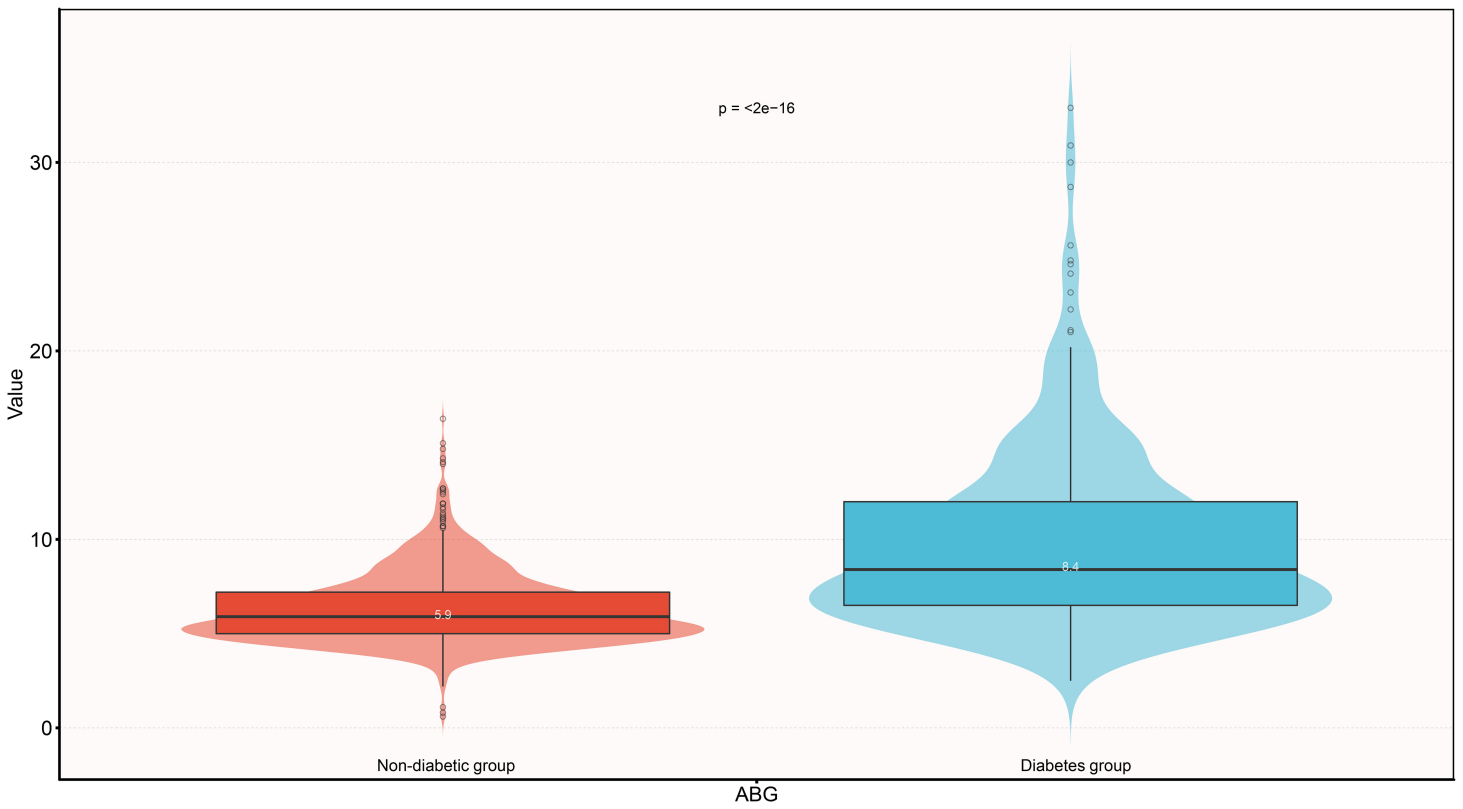
Violin diagram showing ABG baseline characteristics according to whether there is diabetes or not. ABG, admission blood glucose.

**Table 2** shows information on the baseline characteristics of the study population, and we found that the vast majority of the study population in the current cohort progressed from an acute exacerbation of chronic HF to a decompensated state, with only a small proportion of the population categorized as having new-onset ADHF. After grouping according to whether the subjects were diabetic, we also found that compared to the non-diabetic group, diabetic subjects at baseline had significantly higher proportions of hypertension, CHD, heart failure with reduced ejection fraction (LVEF < 50%), underwent SGLT-2 treatment, NYHA class IV, and higher levels of SBP, ABG, HbA1c, WBC, RBC, PLT, ALB, GGT, Cr, and TG, as well as lower levels of LVEF and HDL-C. Notably, there was a considerable difference in baseline ABG between the two groups (standardized difference value = 97%, **Figure 3**). Additionally, it is also important to mention that diabetic factors may lead to higher incidence of hypertension [Odds ratio (OR): 2.45, 95% confidence interval (CI): 1.91-3.14)], CHD (OR: 2.35, 95%CI: 1.82-3.02), and lower incidence of heart failure with preserved ejection fraction (OR:0.71, 95%CI: 0.56-0.93). In terms of medication, there was no significant difference between the diabetic and non-diabetic groups in the use of diuretic, ACEI/ARB/ARNI, beta-blockers, digitalis, and corticosteroids.

**Table 2:** Summarize the baseline characteristics of the study population according to whether they are complicated with diabetes or not.

	Non-diabetic group	Diabetes	Standardized Difference (95% CI), %	OR (95% CI)	*P*-value
No. of subjects	1089	373			
Age (years)	70.00 (60.00-79.00)	69.00 (60.00-77.00)	5 (-7, 16)		0.090
Gender					0.097
Female	477 (43.80%)	145 (38.87%)			
Male	612 (56.20%)	228 (61.13%)			
Comorbidities					
Hypertension (n,%)	386 (35.45%)	214 (57.37%)		2.45 (1.91, 3.14)	<0.001
Cerebral infarction (n,%)	163 (14.97%)	70 (18.77%)		1.31 (0.95, 1.80)	0.084
CHD (n,%)	284 (26.08%)	169 (45.31%)		2.35 (1.82, 3.02)	<0.001
NYHA classification (n,%)				1.53 (1.19, 1.97)	<0.001
III	777 (71.35%)	231 (61.93%)			
IV	312 (28.65%)	142 (38.07%)			
ADHF seizure characteristics				0.83 (0.57, 1.88)	0.297
Acute decompensation in CHF	928 (85.22%)	326 (87.40%)			
New‐onset	161 (14.78%)	47 (12.60%)			
LVEF category				0.71 (0.56, 0.93)	0.009
HFrEF	527 (50.82%)	204 (58.96%)			
HFpEF	510 (49.18%)	142 (41.04%)			
Anti-heart failure treatment					
Diuretic	1051 (96.51%)	360 (96.51%)		1.00 (0.51, 2.07)	0.997
CEI/ARB/ARNI	614 (56.38%)	230 (61.66%)		1.24 (0.97, 1.59)	0.075
Beta-blockers	820 (75.30%)	287 (76.94%)		1.09 (0.82, 1.46)	0.522
Digitalis	493 (45.27%)	166 (44.50%)		0.97 (0.76, 1.24)	0.797
SGLT-2	67 (6.15%)	96 (25.74%)		5.28 (3.51, 7.54)	<0.001
Corticosteroids therapy					
Spirolactone	987 (90.63%)	325 (87.13%)		0.70 (0.48, 1.03)	0.054
Adrenaline	133 (12.21%)	41 (10.99%)		0.89 (0.60, 1.30)	0.530
Glucocorticoid	109 (10.01%)	41 (10.99%)		1.11 (0.74. 1.64)	0.589
SBP (mmHg)	127.16 (23.61)	130.43 (26.70)	13 (1, 25)		0.026
DBP (mmHg)	75.58 (15.57)	76.17 (16.17)	4 (-8, 15)		0.530
ABG (mmol/L)	5.90 (5.00-7.20)	8.40 (6.50-12.00)	97 (85, 109)		<0.001
HbA1c	5.70 (0.39)	7.51 (1.62)	154 (139, 168)		<0.001
WBC (×10^9^/L)	5.94 (4.80-7.61)	6.80 (5.41-8.80)	30 (18, 42)		<0.001
RBC (×10^12^/L)	4.05 (0.76)	4.18 (0.84)	16 (5, 28)		0.005
HGB (g/L)	124.00 (111.00-137.00)	126.00 (110.00-139.00)	8 (-4, 20)		0.342
PLT (×10^9^/L)	159.50 (124.00-205.00)	174.00 (133.00-229.00)	24 (12, 36)		<0.001
ALB (g/L)	35.43 (5.10)	34.67 (4.91)	15 (3, 27)		0.013
ALT (U/L)	21.00 (13.00-37.00)	22.00 (14.00-41.00)	6 (-6, 18)		0.199
AST (U/L)	26.00 (20.00-39.00)	24.00 (18.00-40.00)	7 (-5, 19)		0.081
GGT (U/L)	40.00 (24.00-70.25)	47.00 (26.00-94.25)	20 (8, 32)		0.002
Cr (umol/L)	85.00 (66.00-116.00)	96.00 (72.00-145.00)	31 (19, 43)		<0.001
TG (mmol/L)	1.10 (0.86-1.46)	1.33 (1.04-1.78)	28 (15, 41)		<0.001
TC (mmol/L)	3.70 (3.11-4.38)	3.78 (3.14-4.39)	7 (-5, 20)		0.354
HDL-C (mmol/L)	1.03 (0.30)	0.96 (0.27)	24 (11, 36)		<0.001
LDL-C (mmol/L)	2.37 (0.85)	2.42 (0.92)	6 (-7, 19)		0.339
NT-proBNP (pmol/L)	3685.00 (2131.00-5688.00)	3651.00 (2159.00-6003.00)	5 (-7, 17)		0.588

CHD, coronary heart disease; NYHA, New York Heart Association; LVEF, left ventricular ejection fraction; SBP, systolic blood pressure; DBP, diastolic blood pressure; TG, triglyceride; TC, total cholesterol; HDL-C, high-density lipoprotein cholesterol; LDL-C, low-density lipid cholesterol; Cr, creatinine; WBC, white blood cell count; RBC, red blood cell count; HGB, hemoglobin; PLT, platelet count; ALT, alanine aminotransferase; AST, aspartate aminotransferase; GGT, glutamyl transpeptidase; ALB, albumin; NT-proBNP, N-Terminal Pro-Brain Natriuretic Peptide; ABG, admission blood glucose; SGLT-2, sodium-dependent glucose transporters 2; HFrEF, heart failure with reduced ejection fraction (LVEF < 50%); HFpEF, heart failure with preserved ejection fraction (LVEF ≥ 50%); ACEI, angiotensin-converting enzyme inhibitors; ARB, angiotensin receptor inhibitors; ARNI, angiotensin receptor neprilysin inhibitors; OR, odds ratio; CI, confidence interval.

### Association between ABG and 30-day mortality in ADHF Patients with and without diabetes

Five progressively adjusted Cox regression models were constructed to assess the relationship between ABG and 30-day mortality in ADHF patients with and without diabetes (**Table 3**). In the unadjusted model, ABG was positively associated with 30-day mortality, with hazard ratios (HRs) of 1.36 in non-diabetic ADHF patients and 1.20 in diabetic ones. In the first adjusted model (Model 1), considering the potential impact of age, gender, NT-proBNP, routine blood parameters (WBC, RBC, Hb, PLT), and lipid parameters (TG, HDL-C, LDL-C), ABG remained positively associated with 30-day mortality in both groups. The results of the second model (Model 2), which further adjusted for Alb, AST, GGT, Cr, LVEF, were similar to Model 1. Model 3 was further adjusted for NYHA classification, SBP, DBP, hypertension, cerebral infarction and CHD on the basis of Model 2, and the results showed that ABG remained positively correlated with 30-day mortality in patients with ADHF in both diabetic and non-diabetic groups. The fifth model (Model 4), our final model, included all non-collinear covariates except TC and ALT, showing that with every 1 mmol/L increase in ABG, the 30-day mortality risk increased by 76% [HR 1.76, 95% CI: 1.02, 3.04] in diabetic ADHF patients and by 33% (HR 1.33, 95% CI: 1.06, 1.66) in non-diabetic ones. The relative 30-day mortality risk was higher in diabetic ADHF patients compared to non-diabetics (HR: 1.76 vs 1.33). Based on Model 4 results, we further calculated the E-values for the association between ABG and 30-day mortality in ADHF patients with and without diabetes, with point estimates of 2.92 and 1.99, respectively.

**Table 3:** Multivariable Cox regression analysis of the association between ABG and 30-day mortality in patients with ADHF.

	HR (95% CI)	
	Diabetes group	Non-diabetic group
No. of subjects	373	1089
30-day Mortality, n (%)	20 (5.36%)	33 (3.03%)
Unadjusted Model	1.36 (1.20, 1.54)	1.20 (1.14, 1.26)
Adjust Model 1	1.17 (1.08, 1.27)	1.16 (1.05, 1.39)
Adjust Model 2	1.26 (1.11, 1.43)	1.19 (1.08, 1.44)
Adjust Model 3	1.65 (1.11, 2.44)	1.37 (1.09, 1.73)
Adjust Model 4	1.76 (1.02, 3.04)	1.33 (1.06, 1.66)

HR, hazard ratios; CI, confidence interval; ADHF, acute decompensated heart failure; other abbreviations as in **Table 1**.

Model 1 adjusted for age, sex, NT-proBNP, TG, HDL-C, LDL-C, WBC, RBC, Hb, PLT.

Model 2 adjusted for age, sex, NT-proBNP, TG, HDL-C, LDL-C, WBC, RBC, Hb, PLT, ALB, AST, GGT, Cr, LVEF.

Model 3 adjusted for age, sex, NT-proBNP, TG, HDL-C, LDL-C, WBC, RBC, Hb, PLT, ALB, AST, GGT, Cr, LVEF, NYHA, SBP, DBP, hypertension, cerebral infarction and CHD.

Model 3 adjusted for age, sex, NT-proBNP, TG, HDL-C, LDL-C, WBC, RBC, Hb, PLT, ALB, AST, GGT, Cr, LVEF, NYHA, SBP, DBP, hypertension, cerebral infarction, CHD, HbA1c, etiology of ADHF, ADHF seizure characteristics, anti-heart failure treatment and corticosteroids therapy.

### Dose-response relationship

**Figure 4:**
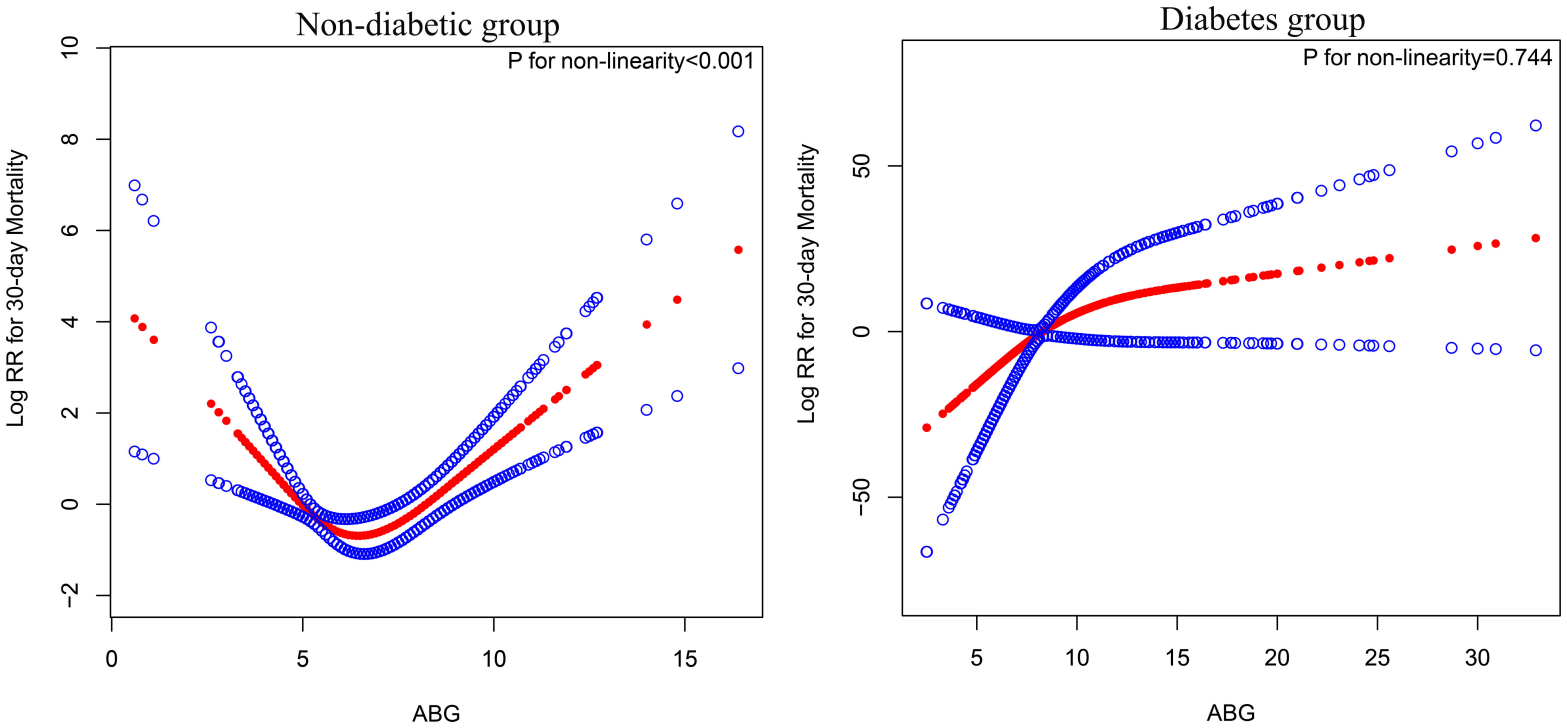
Fitting the dose-response relationship between ABG and 30-day mortality in ADHF patients with 4 knots restricted cubic spline. ABG, admission blood glucose; ADHF, acute decompensated heart failure. Adjusted for age, gender, NT-proBNP, TG, HDL-C, LDL-C, WBC, RBC, Hb, PLT, ALB, AST, GGT, Cr, LVEF, NYHA, SBP, DBP, hypertension, cerebral infarction and CHD.

Using RCS, we further analyzed the dose-response relationship between ABG and 30-day mortality in ADHF patients, grouped according to diabetes diagnosis, with variable adjustments following the Model 4 scheme from **Table 3**. As depicted in **Figure 4**, a U-shaped association between ABG and 30-day mortality was observed in non-diabetic ADHF patients (*P* for non-linearity < 0.001), with the lowest risk of mortality around 5-7 mmol/L ABG. In diabetic ADHF patients, a linear relationship was noted (*P* for non-linearity = 0.744), showing a linear increase in mortality with rising ABG levels (**Figure 4**).

### Subgroup analysis

Exploratory subgroup analyses were conducted to investigate the differences in the association between ABG and 30-day mortality in ADHF patients with and without diabetes across various populations, stratified by age, gender, and comorbidities. Across multiple subgroups, we did not observe any significant interactions (**Table 4**; all *P*-interaction > 0.05), and these findings suggested that the current research results were relatively stable.

**Table 4:** Stratified analysis showed the relationship between ABG and 30-day mortality in patients with ADHF in different age, sex, NYHA class, LVEF and whether combined with hypertension/Cerebral infarction/CHD.

Subgroup	HR (95% CI)	
	Diabetes group	Non-diabetic group
Sex		
Male	2.03 (1.07, 3.87)	1.48 (1.12, 1.95)
Female	1.56 (0.87, 2.79)	1.10 (0.75, 1.62)
*P* for interaction	0.1975	0.1969
Age (years)		
20-70	1.53 (1.02, 2.30)	1.53 (1.09, 2.14)
71-96	1.40 (1.03, 1.90)	1.14 (0.83, 1.56)
*P* for interaction	0.6721	0.2098
Hypertension		
Yes	1.84 (1.11, 3.05)	1.46 (0.99, 2.14)
No	1.43 (0.87, 2.34)	1.27 (0.97, 1.67)
*P* for interaction	0.1263	0.5600
Cerebral infarction		
Yes	2.03 (0.92, 4.50)	1.61 (0.95, 2.74)
No	1.68 (1.00, 2.84)	1.28 (0.99, 1.64)
*P* for interaction	0.1210	0.4265
CHD		
Yes	1.69 (0.92, 3.09)	0.68 (0.38, 1.21)
No	2.64 (0.93, 7.54)	1.70 (1.26, 2.31)
*P* for interaction	0.2063	0.0810

Abbreviations in **Table 2**.

Models adjusted for the same covariates as in model 4 (**Table 3**), except for the stratification variable.

### ROC analysis

The accuracy of ABG in predicting 30-day mortality in ADHF patients with and without diabetes was further assessed using ROC analysis. As shown in **Table 5**, the AUC for ABG in predicting 30-day mortality was 0.7124 in non-diabetic ADHF patients, with an optimal threshold of 7.05 mmol/L. In diabetic ADHF patients, the AUC was 0.8751 with a predictive threshold of 13.95 mmol/L. Compared to the non-diabetic group, ABG demonstrated much higher accuracy in predicting 30-day mortality in diabetic ADHF patients, with a significantly higher predictive threshold.

**Table 5:** ROC analysis of the predictive value of ABG for 30-day mortality in ADHF patients with or without diabetes.

	AUC	95%CI low	95%CI upp	Best threshold	Specificity	Sensitivity
ABG (Non-diabetic)	0.7124	0.6113	0.8135	7.0500	0.7386	0.6364
ABG (Diabetes)	0.8751	0.8003	0.9500	13.9500	0.8669	0.7500

ABG, admission blood glucose; AUC, area under the curve; ADHF, acute decompensated heart failure.

### Mediation analysis

Following the establishment of a longitudinal association between ABG and 30-day mortality in ADHF patients, mediation models were used to analyze the effects of WBC, GGT, and ALB in mediating this relationship in patients with and without diabetes. Mediation analysis results (**Table 6**) revealed that among the three pathways, inflammation, oxidative stress, and nutrition, only inflammation significantly mediated the ABG-related 30-day mortality (*P*-value of proportion mediated < 0.05). Specifically, inflammation accounted for approximately 11.15% of the mediation effect on the ABG-related 30-day mortality in diabetic ADHF patients, and about 8.77% of the effect in non-diabetic ADHF patients.

**Table 6:** Mediated analysis was performed to explore the roles of inflammation, oxidative stress and nutritional pathways in the association between ABG and the 30-day mortality rate in ADHF patients.

Mediator	Total effect	Mediation effect	Direct effect	PM(%)	*P*-value of PM
Diabetes group					
WBC	0.038 (0.022, 0.068)	0.004 (0.000, 0.012)	0.034 (0.018, 0.063)	11.15	0.028
GGT	0.038 (0.022, 0.068)	-0.001(-0.005,0.024)	0.039 (0.023, 0.071)	0.6	0.730
ALB	0.038 (0.002, 0.068)	0.002 (-0.000, 0.006)	0.036 (0.020, 0.062)	4.92	0.072
Non-diabetic group	0.016 (0.009, 0.024)	0.000 (-0.001, 0.000)	0.017 (0.010, 0.025)	0.65	0.550
WBC	0.016 (0.008, 0.023)	0.002 (0.000, 0.029)	0.014 (0.007, 0.021)	8.77	0.004
GGT	0.016 (0.008, 0.023)	0.000 (-0.000, 0.001)	0.016 (0.009, 0.024)	0.38	0.496
ALB	0.016 (0.008, 0.023)	0.000 (-0.000, 0.001)	0.016 (0.008, 0.023)	1.01	0.636

PM, propotion mediate; other abbreviations as in **Table 1**.

Model adjusted for the same covariates as in model 3 (**Table 3**), except for the mediator variable.

## Discussion

In this study, using the JX-ADHF1 study cohort, we discovered that ABG was an independent predictor of 30-day mortality in Chinese patients with ADHF. ABG was particularly applicable for assessing 30-day mortality risk and predicting death events in ADHF patients with diabetes, more so than in those without.

Elevated blood glucose levels are very common in patients presenting with acute symptoms (7, 16, 17), and this stress response adversely impacts various medical conditions, including AHF (16), acute coronary syndromes (9, 34), acute ischemic stroke (35), acute pancreatitis (36), sepsis (13, 37), and various critical illnesses (8, 38). In the context of AHF, the relationship between ABG and 30-day mortality remains controversial, with contradictory findings in completed studies. For instance, studies by Professor Mebazaa A and Professor Sud M in independent European and American cohorts indicated that ABG was independently related to 30-day mortality in AHF patients (17, 18). Conversely, research by Professor Kosiborod M and Professor Cox ZL in independent American elderly cohorts suggested no correlation between ABG and 30-day mortality in AHF patients (16, 39). The studies by Professors Mebazaa and Sud included a broader range of ages and samples from multiple countries in Europe and America, potentially offering greater representativeness in their findings. In our current study, based on the adult ADHF cohort from Jiangxi, China, our evidence supported a correlation between ABG and 30-day mortality in ADHF patients, with no significant differences observed in the age subgroup analysis between the elderly and other age groups. Furthermore, we assessed this association in diabetic and non-diabetic populations, finding that, after adjusting for confounding factors, ADHF patients with diabetes had a relatively higher 30-day mortality risk compared to those without (HR: 1.65 vs 1.37). Notably, the unadjusted model indicated a higher correlation between ABG and 30-day mortality in non-diabetic ADHF patients, suggesting that in patients with a known diagnosis of diabetes, hyperglycemia might receive better monitoring and treatment from the onset of hospital admission.

The predictive value of ABG for 30-day mortality in AHF patients has been reported in previous studies, such as that by Professor Mebazaa A (17), but there remains a lack of sufficient evidence to clearly establish ABG’s utility in the short-term prognosis of ADHF patients. Retrospective studies have provided evidence of ABG’s predictive value for short-term adverse outcomes in other diseases. As summarized in **Supplementary Table 3**, in the context of severe trauma, studies from Germany and Switzerland have shown ABG’s utility in predicting in-hospital shock (AUC=0.62) and in-hospital mortality (AUC=0.788), respectively (40, 41). Furthermore, in predicting in-hospital major adverse events for acute and critical illnesses, AUC values for myocardial infarction (42), spontaneous intracerebral hemorrhage (43), acute stroke (44), and intensive care unit admissions (45) hover around 0.7. These findings suggest that ABG is a valuable predictor of short-term adverse outcomes in critical illnesses; however, it is important to note the need for stratification by diabetes, given the general disparity in ABG levels between diabetic and non-diabetic patients (7, 16, 17). In our study, to clarify ABG’s predictive value for 30-day mortality in ADHF patients, we conducted ROC analysis after stratifying by diabetes status. Our results showed that ABG’s AUC for predicting 30-day mortality was 0.8751 in diabetic ADHF patients and 0.7124 in non-diabetics, indicating higher accuracy in diabetic patients.

After establishing ABG’s predictive value and risk assessment capability for 30-day mortality in ADHF patients, it is crucial to estimate clinically applicable threshold values. In a previous study by Mebazaa A, the ABG threshold for estimating 30-day mortality in non-diabetic ADHF patients was found to be 7 mmol/L, and 10 mmol/L in diabetics (17). In our study, through RCS, we evaluated the dose-response relationship curve between ABG and 30-day mortality in ADHF patients, revealing a U-shaped association in non-diabetics, with the lowest risk around 5-7 mmol/L. We observed a linear positive relationship in diabetics without a significant threshold or saturation effect point. ROC analysis further identified predictive thresholds for 30-day mortality, which were 7.05 mmol/L for non-diabetics and 13.95 mmol/L for diabetics, aligning with Mebazaa A’s findings and suggesting higher thresholds in diabetic patients (17). Additionally, considering ABG’s predictive thresholds in other diseases (**Supplementary Table 3**) (40–46), we recommend closer monitoring and intensified endocrine treatment for critical illness patients with ABG exceeding 10 mmol/L (7, 47–49). For ADHF patients in China, those with diabetes showing ABG levels above 13.95 mmol/l and those without diabetes exceeding 7.05 mmol/l should be closely monitored for potential short-term severe adverse outcomes. Strengthening surveillance and optimizing glycemic management strategies are advised in these scenarios.

Our findings broadly confirmed that ABG can serve as a risk biomarker for 30-day mortality in ADHF patients. However, it cannot be excluded that stress hyperglycemia acts as a mediator to produce adverse effects by mediating other important pathways, because stress hyperglycemia occurs through highly complex interactions of regulatory hormones such as cytokines, cortisol, growth hormone, and catecholamines (50–52). The exact mechanisms of interaction between ABG and adverse outcomes remain unclear; however, stress hyperglycemia exacerbating inflammation, cytokine, and oxidative stress responses, potentially forming a vicious cycle leading to further hyperglycemia, should be noted (53–55). This cycle might be a key factor in the poor short-term prognosis of high-ABG ADHF patients. From a cardiac perspective, high glucose levels directly promote myocardial cell calcium metabolic disorder, increased NF-κB levels, and upregulation of matrix metalloproteinases, leading to apoptosis and progressive remodeling (17, 56). High glucose levels also might cause abnormal increases in circulating free fatty acid concentrations, raising the risk of arrhythmias (57). Additionally, our study assessed the mediating roles of inflammation (27, 53, 54), oxidative stress (28, 53, 54), and nutritional pathways (29). We found that only inflammation significantly mediated ABG-related 30-day mortality in ADHF patients, accounting for 11.15% in diabetics and 8.77% in non-diabetics. This highlights the critical assessment value of inflammation in acute exacerbation diseases, where inflammation not only independently affects adverse outcomes in ADHF patients but also indirectly exacerbates these outcomes through ABG (58).

The short-term adverse events in patients with ADHF have always been among the most challenging issues in clinical settings, as hospitalization for ADHF itself signifies a poor prognosis (1, 2). In the present study, we validated the prognostic value of a simple yet effective marker, the ABG, for predicting short-term adverse events in patients with ADHF. It is important to mention that in addition to the high predictive power of ABG for poor prognosis in patients with ADHF, the measurement of this index is quite simple and is routinely performed in both outpatient and inpatient clinical settings. Hence, ABG emerges as a promising tool for risk stratification and prognostic assessment in ADHF patients, offering valuable additional information to clinicians. It is crucial to emphasize that, although this study demonstrated the value of ABG in predicting the short-term mortality of ADHF patients, ABG should not be used in isolation in the clinical treatment decision-making process. It should always be integrated with the clinical judgment of professional physicians, adopting a comprehensive approach that primarily guides the use of validated risk assessment tools in the clinical decision-making process, guided by the personal professional knowledge of clinicians. The rational and correct application of risk assessment tools will aid clinicians in identifying the most appropriate management pathways, thereby promoting effective and safe clinical decision-making (59, 60). Furthermore, as glucose markers in patients may change during treatment, close monitoring and frequent reassessment are essential for subsequent clinical decisions to ensure that the patient’s condition is moving in the right direction (61). It should also be noted that the application of ABG in prognostic evaluation and risk stratification should be accompanied by efforts to identify the triggers of ADHF episodes, as recognizing these triggers occupies a central place in the management and disposition of ADHF. Clinical practitioners should always consider and fully address all factors that could potentially worsen the patient’s condition. Finally, in conjunction with the findings of the current study, for future research we suggest focusing on stress-related parameters in acute exacerbation diseases and testing the joint role of combined ABG and inflammatory factors.

## Strengths and limitations

The main strength of this study lies in its novel revelation of the relationship between ABG and short-term adverse outcomes in Chinese ADHF patients, with comprehensive analysis stratified by diabetes status. This study is the first to identify a U-shaped association between ABG and 30-day mortality in non-diabetic ADHF patients and to determine the ABG threshold for ADHF patients with and without diabetes. Additionally, it’s the first to identify the significant role of the inflammation pathway in ABG-related 30-day mortality in ADHF patients through mediation analysis. Overall, our study fills a gap in the understanding of the relationship between ABG and adverse outcomes in ADHF patients in the Chinese population and significantly expands the understanding of stress hyperglycemia in predicting adverse outcomes, particularly with regard to diabetes stratification.

However, there are limitations to this study: (i) The lack of continuous blood glucose monitoring data during the hospitalization of ADHF patients, which might provide more advantageous risk stratification information compared to ABG. (ii) Another limitation, due to the retrospective and non-interventional nature of the study, is the inability to obtain blood glucose information of the subjects at the end of the 30-day follow-up. (iii) Our study population mainly comes from Jiangxi, China, and caution is advised when other ethnic groups or other regions in China consider our findings. (iv) Due to the retrospective design, we couldn’t fully account for all disease-related measurement information; hence, despite significant efforts to control confounding factors, some residual confounding is inevitable (62). As a supplementary explanatory approach, we further calculated the E-value based on Model 3 results (26), suggesting relative stability of our findings as the high estimate HRs of 2.92 and 1.99 for 30-day mortality are unlikely to be achieved by most confounders. (v) Due to the retrospective design and non-interventional principle, we couldn’t quantitatively analyze the impact of endocrine and anti-inflammatory treatments on the outcomes in ADHF patients after admission. (vi) Finally, it is also important to mention that retrospective designs rely on pre-existing data are more susceptible to a variety of biases, including selection bias and information bias, which limits the scope and depth of the study.

## Conclusion

Our study confirms ABG as an independent predictor of 30-day mortality in ADHF patients. Compared to non-diabetic subjects, ABG may be more suitable for assessing 30-day mortality risk and predicting death events in ADHF patients with diabetes. For ADHF patients with/without diabetes, our evidence suggests that when ABG exceeds 13.95/7.05 mmol/l, respectively, physicians should be alert and closely monitor any changes in patient conditions. Prompt adjustments to existing treatment plans, particularly in endocrine therapy and anti-inflammatory treatment, are recommended.

## Data availability statement

The raw data supporting the conclusions of this article will be made available by the authors, without undue reservation.

## Ethics statement

The studies involving humans were approved by the ethics review committee of Jiangxi Provincial People’s Hospital. The studies were conducted in accordance with the local legislation and institutional requirements. The participants provided their written informed consent to participate in this study.

## Author contributions

JH: Formal analysis, Investigation, Validation, Writing – original draft. HY: Formal analysis, Investigation, Validation, Writing – original draft. MY: Formal analysis, Investigation, Software, Validation, Writing – original draft. CY: Investigation, Writing – review & editing. JQ: Investigation, Writing – review & editing. GX: Data curation, Investigation, Software, Writing – review & editing. GS: Data curation, Investigation, Writing – review & editing. MK: Formal analysis, Investigation, Software, Validation, Writing – original draft. YZ: Conceptualization, Data curation, Investigation, Methodology, Project administration, Supervision, Writing – review & editing.
