# Supplementary material for: Admission blood glucose and 30-day mortality in patients with acute decompensated heart failure: prognostic significance in individuals with and without diabetes
Source: Front Endocrinol (Lausanne). 2024 Jul 5;15:1403452. doi: 10.3389/fendo.2024.1403452 (PMC11257984; doi:10.3389/fendo.2024.1403452)
Supplement: Supplementary file 1 [file DataSheet_1.docx]

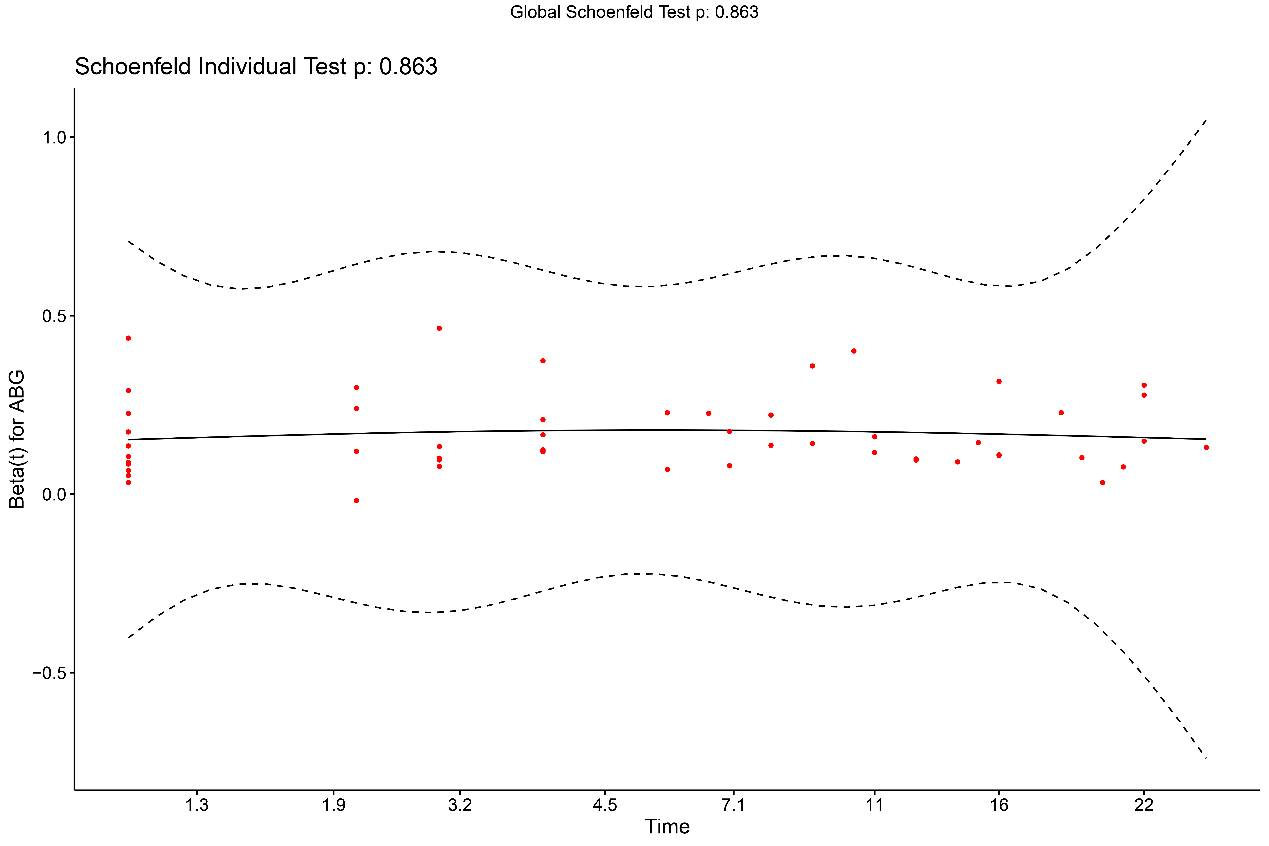


**Supplementary Figure 1**: Schoenfeld residual plot of ABG over time with 30-day mortality in ADHF patients as the dependent variable. The p-value of Schoenfeld Residuals Test result is larger than 0.05 which indicated that ABG is not a time dependent variable and can be analyzed by Cox Proportional Hazards Model.
